# Impaired Hepatitis B and COVID-19 vaccination responses show strong concordance in hemodialysis patients with end stage renal disease

**DOI:** 10.1186/s40001-025-02274-3

**Published:** 2025-01-16

**Authors:** Karsten Lürken, Anna Meinecke, Luis A. Manthey, Anne Cossmann, Metodi V. Stankov, Frank Klawonn, Anna Zychlinsky Scharff, Sandra Steffens, Alexandra Dopfer-Jablonka, Frank Müller, Georg M. N. Behrens, Christine Happle

**Affiliations:** 1 Dialysis Centre Eickenhof, Langenhagen, Germany; 2 Department of Rheumatology and Immunology, Hannover Medical School, Hannover, Germany; 3 German Centre for Infection Research (DZIF), Partner Site Hannover-Braunschweig, Hannover, Germany; 4 Institute of Information Engineering, Ostfalia University of Applied Sciences, Wolfenbüttel, Germany; 5 Biostatistics Research Group, Helmholtz-Center for Infection Research, Braunschweig, Germany; 6 Department of Pediatric Pneumology, Allergology, and Neonatology, Hannover Medical School, Hannover, Germany; 7 Department of General Practice, University Medical Center Göttingen, Göttingen, Germany; 8 Department of Family Medicine, College of Human Medicine, Michigan State University, Grand Rapids, MI USA; 9 CiiM – Centre for Individualized Infection Medicine, Hannover, Germany; 10 Biomedical Research in End Stage and Obstructive Lung Disease/BREATH Hannover, German Centre for Lung Research (DZL), Hannover, Germany; 11 Department of Pediatric Oncology and Hematology, Hannover Medical School, Hannover, Germany

**Keywords:** Hemodialysis, HBV, SARS-CoV-2, Vaccination response

## Abstract

**Background:**

Patients with end stage renal disease (ESRD) undergoing hemodialysis are at increased risk for infection and impaired vaccination responses. We analyzed overlap and influencing factors of vaccination responses against severe acute respiratory syndrome corona virus disease 2 (SARS-CoV-2) and Hepatitis B virus (HBV).

**Methods:**

SARS-CoV-2 and HBV vaccination response was assessed in a cohort of German ESRD hemodialysis patients. Anti-HBs- and SARS-CoV-2 anti-S-IgG were analyzed by ELISA. Demographic and clinical data were extracted from clinical files.

**Results:**

Sixty-four patients with complete information on HBV and SARS-CoV-2 vaccination responses were included. More than one-third (35.4%) of non-responders upon HBV vaccination were identified. Unresponsiveness after HBV and poor response after SARS-CoV-2 vaccination showed strong overlap, and overall, 70.3% of patients were classified into concordant HBV/SARS vaccination response groups. HBV vaccination non-responsiveness, but not poor SARS-CoV-2 post-vaccination immunity was associated with obesity, while poor SARS-CoV-2 vaccination responses were associated increased age.

**Conclusion:**

Our findings confirm previous reports on impaired vaccination response in hemodialysis patients and show that post-vaccination humoral responses against SARS-CoV-2 and HBV display strong overlap in this vulnerable patient group. These results may help to adapt vaccination strategies in this highly vulnerable population.

*Trial registration*: German Clinical Trial Registry, DRKS00021152.

**Supplementary Information:**

The online version contains supplementary material available at 10.1186/s40001-025-02274-3.

## Introduction

Acquired infections are a leading cause of death patients with end stage renal disease (ESRD) patients [1]. ESRD patients, particularly those on hemodialysis, are at increased risk for viral infections and susceptible to severe disease courses upon infection with severe acute respiratory syndrome corona virus disease 2 (SARS-CoV-2) and Hepatitis B virus (HBV [2–4]). Due to the uremic milieu, malnutrition, and chronic inflammation with impaired T and antigen-presenting cell function, they show a significantly impaired host defense against infection with reduced humoral immune response [1, 5–7]. The need for frequent vascular access and medical treatment further increases the risk for viral infections such as with HBV [8]. Also, ESRD patients had impaired post-vaccination immunity with accelerated decline of antibody levels [9–12].

Both SARS-CoV-2 infections and inadequate HBV vaccination responses are associated with increased morbidity and mortality in ESRD patients [13–15], and more frequent booster vaccinations and higher antigen doses are required [12, 16–19]. However, little is known on how HBV vaccination responses relate to those against COVID-19 in ESRD patients, and on the factors influencing vaccination response in this vulnerable population. This is relevant because each vaccine stimulates profoundly different pathways in the immune system. While most HBV vaccines use adjuvants such as aluminium salt and Adjuvant System 04 (AS04) consisting of aluminium hydroxide and monophosphoryl lipid A (MPL) and stimulate Toll-like receptors and NLRP3 inflammasome [20], mRNA vaccines primarily stimulate monocytes, IFN gamma, and IL-1, amplified by certain lipids used in vaccine formulations incorporating *N*^1^-methyl-pseudouridine-modified RNA to reduce activation of Toll-like receptor signaling [21, 22].

Here, we assessed the humoral immune response after HBV and mRNA COVID-19 vaccination in ESRD patients undergoing hemodialysis. We show that response patterns against HBV and COVID-19 vaccinations are related and identify factors associated with poor vaccination responses against these pathogens.

## Methods

### Study design and sample collection

This work was performed as part of the CoCo (Covid-19-Contact) study (German Clinical Trial Registry, DRKS00021152), which has been described in detail elsewhere [16, 23, 24]. For this cross-sectional study, ESRD patients receiving regular dialyses at the dialysis center Eickenhof (Langenhagen, Germany) aged 18 years or older were enrolled as a convenience sample. Participants were recruited from February 2021 onwards. After obtaining written informed consent, heparinized blood samples were drawn from participants, either from arterio-venous fistulas or central venous lines prior to routine dialysis.

### Clinical data collection

Data on case history and treatment of ESRD patients were extracted from routine clinical documentation. Age and body mass index (BMI) were assessed at the time point of SARS-CoV-2 vaccination.

### Vaccination regimens and analysis of immune response

mRNA COVID-19 vaccinations were performed for the entire cohort between February and June 2021 as part of the national COVID-19 vaccination campaign, and immune response against this pathogen were analyzed prospectively. All participants received the standard two-dose regimen of BNT162b2 21 days apart, which was considered a complete immunization. Vaccination response was assessed 21 days after complete COVID-19 vaccination. To rule out acute SARS-CoV-2 infections during the COVID-19 vaccination analysis period, a surveillance of COVID-19 symptoms was performed every 3 days, and all patients were tested by rapid antigen or polymerase chain testing whenever an infection was suspected. The following HBV vaccinations were applied Fendrix (GlaxoSmithKline Biologicals S.A., Rixensart, Belgium) containing 1× 20 µg of HBs-antigen per injection with the adjuvants AS04 and aluminium salt, HBVaxPro (MSD vaccines, Lyon, France; 2 vials (1× 40 µg of HBs-antigen per injection) containing aluminium salt as main adjuvant), and Engerix-B (GlaxoSmithKline Biologicals S.A., Rixensart, Belgium) 2× 20 µg per injection with aluminium salt as main adjuvant). Vaccinations were applied during routine clinical procedures, and complete HBV vaccination was defined after at least three vaccinations. HBV vaccination response, assessed as anti-HBs IgG levels after complete vaccination, were obtained from patient files. Anti-HBs IgG was determined in routine clinical procedures employing an electrochemiluminescence immunoassay (ECLIA Elecsys Anti-HBs II, Roche diagnostics, Germany) applied following the manufacturer’s recommendations). Non-response to vaccination was defined as anti-HBs-IgG levels <10 U/ml, which was the minimum detection threshold of the assay. To assess the humoral immune response against SARS-CoV-2 prospectively, plasma samples were diluted 1:4000 and analyzed using the Anti-SARS-CoV-2-Spike-Protein-QuantiVac-ELISA IgG (Cat# EI 2606–9601-10G, EUROIOMMUN, Germany) according to the manufacturer’s instructions. Poor response to COVID-19-vaccination was defined as Anti-SARS-CoV-2-Spike-Protein levels below the 25th percentile of all patients (307 BAU/ml), which was close to the cut-off defined by Feng et al. [25] of around 264 BAU/ml to reach around 80% protection from symptomatic COVID-19 after first complete vaccination against this pathogen.

### Ethics statement

Ethical approval of the study was obtained from the internal review board of Hannover Medical School (MHH, approval number 8973_BO-K_2020).

### Statistical analysis

Data were pseudonymized before inclusion into a central database. For statistical analyses, we used Microsoft Excel (Version 2019) R (version 3.6.1), SPSS Statistics (version 20, IBM Corp., Armonk NY, USA), and GraphPad Prism (version 5, Graph Pad Software, San Diego CA, USA). Differences between groups were assessed using Student’s *t*, Mann–Whitney-*U*, or Kruskal–Wallis testing (depending on data structure and distribution), or Fisher´s exact test, and likelihood ratio Chi-square, where appropriate. The direct correlation between immune responses against HBV and COVID vaccination as presented in Suppl. Fig. 2 was calculated by linear regression. A *p* value <0.05 was considered statistically significant.

## Results

**Fig. 1:**
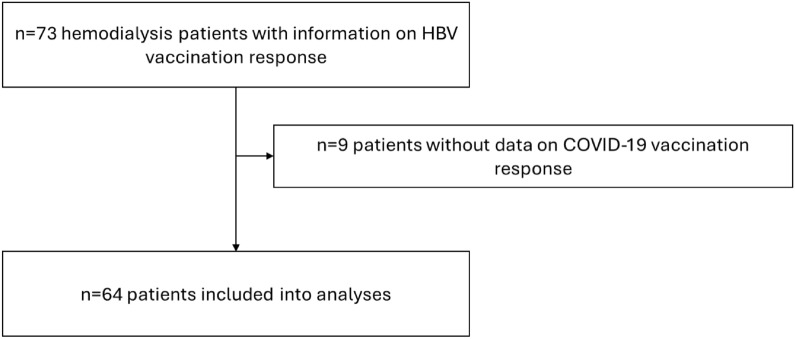
Flowchart of patient inclusion

Sixty-four participants with complete information on COVID-19 and HBV vaccination were included into the analysis. A flowchart of patient inclusion is below (Fig. 1). Twenty-three patients (58.5%) were male, and the median age at COVID-19 vaccination was 71.5 years (IQR 18).

In 92.2% of cases, information on the underlying disease leading to ESRD was available. Nephrosclerosis was the most frequent underlying disease (23.4%), followed by diabetic nephropathy (20.3%), autosomal dominant polycystic kidney disease (14.1%), and IgA nephropathy (10.9%). Further data on underlying diseases are shown in Suppl. Table 1. Fendrix was applied in 47.5%, HBVaxPro in 42.3%, and Engerix-B in 9.8% of HBV vaccinees. To analyze relations between vaccination responses for the two pathogens and determine factors associated with poor vaccination responses, we stratified our cohort into poor- and well-responders. Vaccination response was assessed at a median of 2.7 months (IQR 2.7 months) after the third vaccination. For Fendrix and Engerix-B, up to four injections may be recommended in immunocompromised patient groups, thus we confirmed that anti-HBs immune responses were overlapping between blood withdrawal after the third and fourth injection. Indeed, the concordance of categorization of immune responses after HBV vaccination into non vs. adequate responders between blood withdrawal after the third and fourth vaccination were 97% for Fendrix and 100% for Engerix-B. Also, we verified that there were no significant differences in anti-HBs titers across the three vaccination types (suppl. Figure 1).

For HBV, we categorized 23 individuals with anti-HBs-IgG levels below 10 mIU/mL after the third vaccination as non-responders (35.4%). Median anti-HBs levels after three HBV vaccinations across all patients were 43 mIU/mL (IQR 693). For SARS-CoV-2, the analysis of all plasma samples yielded detectable anti-S1-IgG 3 weeks after complete vaccination with a median of 794 BAU/ml (IQR 4157) across the cohort, and we defined low vaccination response as anti-S-IgG levels below the 25th percentile (<307 BAU/ml, one-quarter of patients).

**Fig. 2:**
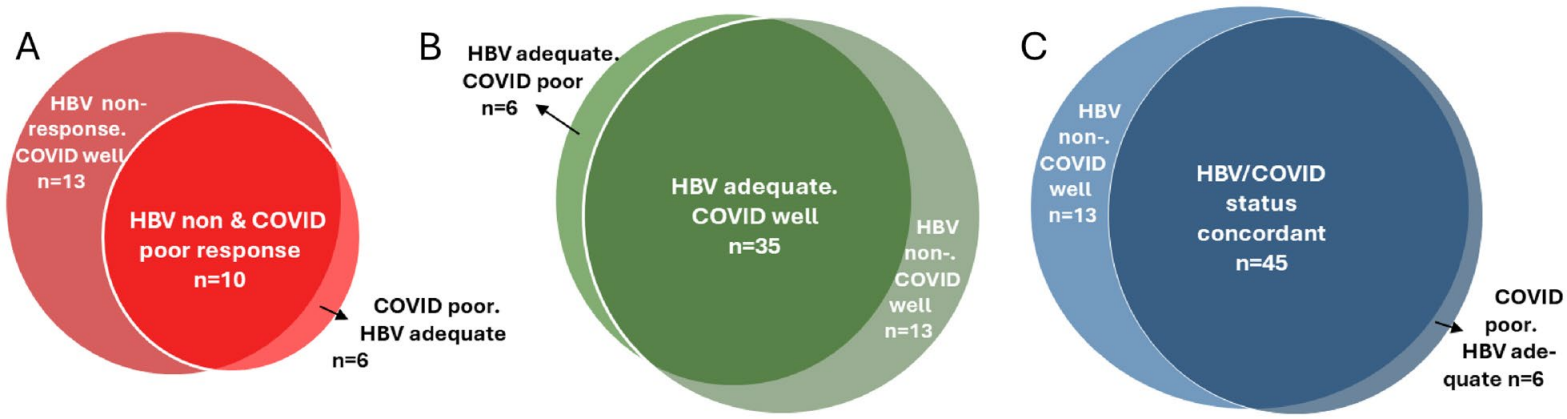
Association of immune response types against HBV- and COVID-19 vaccination. **A** Venn diagram illustrating overlap of HBV non-response and low response after complete COVID-19 vaccination. **B** Concordance and discordance of HBV adequate and SARS-CoV-2 well response. C Overlap of overall vaccination response status against both pathogens

When we compared immune responses after the two vaccinations, we observed a high overlap (Fig. 2). The concordance of HBV vaccination non-responder status with low response after COVID-19 vaccination was considerable (34.4%, Fig. 2A) and a strong overlap of HBV adequate and COVID-19 well response was observed (64.8%, Fig. 2B). Overall, when combining post-vaccination immunity results for both pathogens, 70.3% of patients within the cohort shared response patterns in the sense of displaying non/poor or adequate/well vaccination responses against both pathogens (Fig. 2C).

**Fig. 3:**
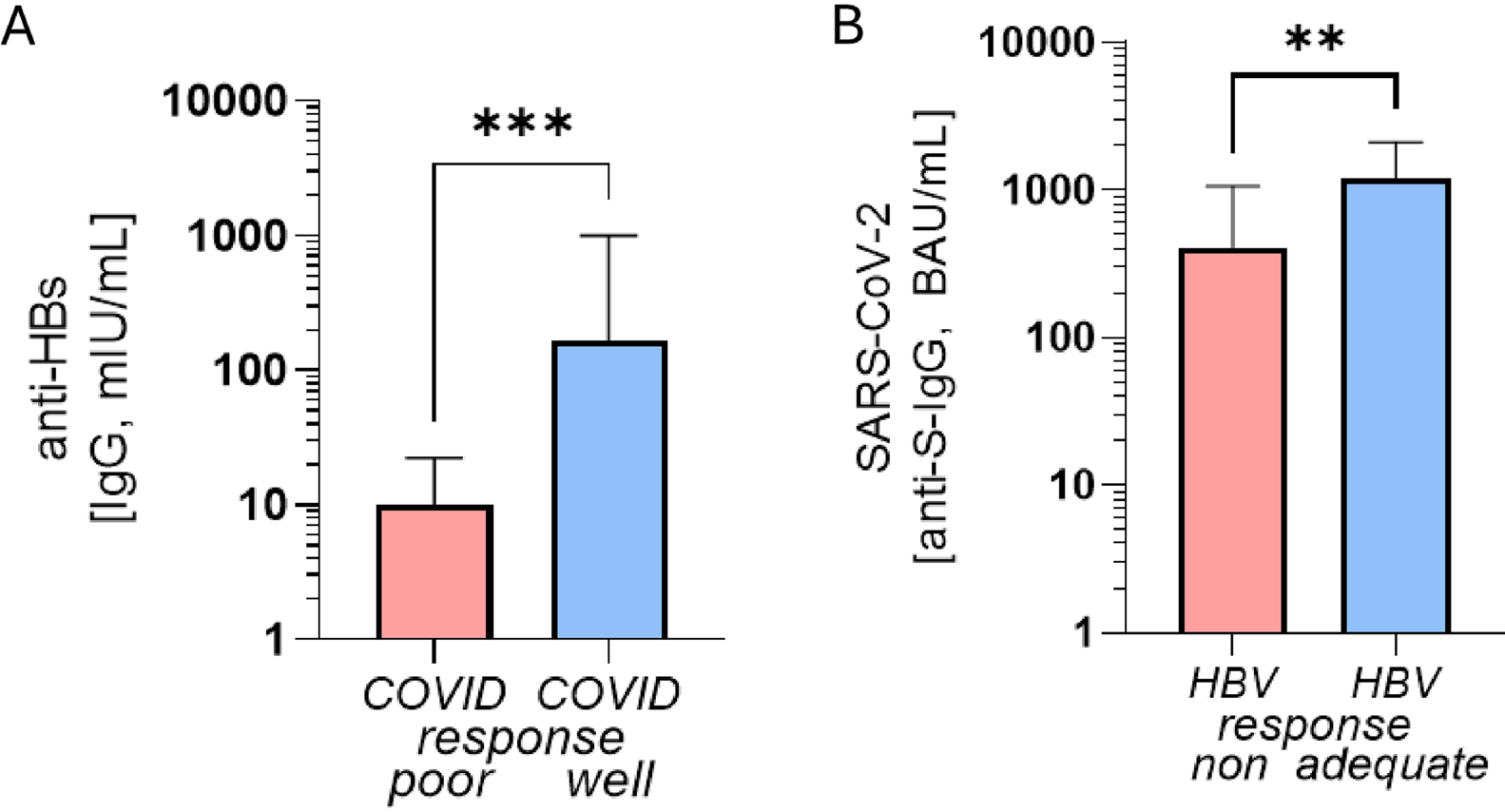
Humoral immune response after HBV vaccination in COVID-19 poor vs. well responders and vice versa. **A** Anti-HBs-IgG titres in well vs. poor responders after COVID-19 immunization. **B** Anti-SARS-CoV-2-S-IgG titres in non- vs. adequate responders after HBV vaccination (bars display median plus IQR, *** *p* < 0.001, ** *p* < 0.01)

Accordingly, ESRD patients with COVID-19 well response displayed significantly higher anti-HBs IgG values than those with low COVID-19 vaccination response (median 10 [IQR 28] vs. 206 [IQR 990] mIU/mL; *p* 0.001, Fig. 3A), and plasma levels of SARS anti-S-IgG in HBV-well-responders were significantly higher than in HBV-non-responders (median 398 [IQR 1057] vs. 1193 [IQR 1516] BAU/mL, *p* 0.001, Fig. 3B). A significant correlation between anti-HBs and anti-S-IgG levels upon complete vaccination occurred (*r*^2^ 0.14, *p* 0.03, Suppl. Fig. 2).

HBV vaccinations preceded COVID-19 vaccination with a mean of 2.2 years (IQR 4.8), and there was no significant difference in the time span between vaccination against the two pathogens in HBV non- vs. adequate responders (median 3.0 [IQR 4.8] vs. 4.8 [IQR 4.3] years, *p* = 0.18).

Next, we analyzed possible associations of vaccination responses with demographic and clinical factors. As shown in Tables 1 and 2, we observed no sex differences in HBV non- vs. well-responders and COVID-19 low- vs. well-responders. While age was not significantly different in the two response groups of HBV vaccinees (Table 1), patients with poor SARS-CoV-2 vaccination responses were significantly older than those with good responses (Table 2). Patients without adequate HBV vaccination response displayed a significant higher obesity rate (defined as body mass index (BMI) >30 kg/m^2^, Table 1), while the rate of obese patients was around 1.5-fold higher, but not significantly increased in COVID-19 poor vs. well responders (Table 2). The rate of angiotensin converting enzyme (ACE) inhibitor, angiotensin receptor blocker, statin, immunosuppression, and vitamin D intake were not significantly different between well and poor responders after vaccination against both pathogens (Table 2). Also, the frequency of diabetes type II was comparable across the vaccination response groups (HBV non-response 34.8% vs. HBV adequate response 26.8%, *p* 0.57; COVID-19 well response 18.8% vs. SARS poor response 33.3%, *p* 0.35).

**Table 1:** Demographic and clinical factors in HBV non vs. adequate responders

	HBV non-response	Adequate HBV response	*p* value
Sex (male)	62.5%	56.1%	0.60
Age	Median 71.0 (IQR 13.0) years	Median 74.0 (IQR 19.0) years	0.53
Obesity	43.9%	14.6%	0.01
ACE inhibitor	34.8%	31.7%	1.00
Angiotensin receptor blocker	13.0%	36.6%	0.08
Statin intake	65.2%	56.1%	0.60
Vitamin D	95.7%	95.1%	1.00
Immunosuppression	13.0%	12.2%	1.00

*ACE* angiotensin converting enzyme

**Table 2:** Demographic and clinical factors in COVID-19 poor vs. well responders

	COVID-19 poor response	COVID-19 well-response	*p* value
Sex (male)	62.5%	58.3%	1.00
Age	Median 76.5 (IQR 18.8) years	Median 71.0 (IQR 18.5) years	0.04
Obesity	31.2%	22.9%	0.52
ACE inhibitor	25.0%	35.4%	0.55
Angiotensin receptor blocker	18.8%	31.2%	0.52
Statin intake	43.8%	64.6%	0.16
Vitamin D	98.3%	95.8%	1.00
Immunosuppression	18.8%	10.4%	0.40

*ACE* angiotensin converting enzyme

## Discussion

Our findings show that post-vaccination humoral responses against COVID-19 and HBV show a strong overlap in ESRD hemodialysis patients and confirm previous results on inhibited vaccination outcomes in this vulnerable patient group. We show that risk factors for low vaccination response differ between HBV and COVID-19. While higher age was associated with poor COVID-19 vaccination responses in our cohort, we found obesity to be linked to impaired HBV humoral immunity post-vaccination.

The overall high rate of impaired HBV vaccination response—more than one-third of our hemodialysis patients were classified as HBV non-responders—is concordant with previous reports on reduced vaccination responses in this population [9–11]. Only around half of ESRD patients undergoing dialysis are reported to develop sustainably protective antibodies after complete HBV vaccination, compared to >90% of healthy persons [6, 7]. Similarly, it has previously been described that ESRD patients show significantly impaired humoral immunity after COVID-19 vaccination and after immunization against other pathogens such as influenza [9, 16, 26]. Reduced HBV and COVID-19 vaccination responses are associated with significant morbidity and mortality in this patient group [12, 16, 17], which makes improved knowledge on risk factors for poor immunization results central in ESRD patient care.

In our cohort, humoral response against both vaccinations showed strong overlap. Approximately 70% of our investigated individuals showed concordance regarding poor- or well-response upon vaccination against the two pathogens. A similar observation was reported by Kolland et al. [27] who showed impaired humoral immunity after COVID-19 vaccination in peritoneal dialysis patients unresponsive to HBV vaccination. By contrast, Nacash et al. [1] compared HBV vs. COVID-19 post-vaccination response in ESRD and found no association between non-response after vaccination. This may be explained by the fact that the vaccination regimens reported in these papers were less robust than ours (at least one HBV vaccination), and an extremely low rate of COVID-19 poor response was defined (15% non-responders in their cohort). Our cohort did not include a single COVID-19 non-responder, as all vaccinated ESRD patients had robustly detectable anti-S1-IgG 3 weeks after complete SARS vaccination. We believe that our stratification of ESRD patients into COVID-19 vaccination low- and high-responders is well suited to identify clinically relevant associations and risk factors: low humoral immunity against COVID-19 is associated with more severe COVID-19 outcomes in healthy persons and renal disease patients [28, 29], and our threshold of 307 BAU/ml for poor response is close to the cut-off reported by Feng et al. [25] of around 264 BAU/ml to induce a ca. 80% protection from symptomatic COVID-19 upon first complete COVID-19 vaccination.

Within our cohort, patient characteristics impacting immune response differed between the two pathogens: on the one hand, higher age was associated with poor COVID-19 vaccination responses. This finding is in line with previous publications illustrating a poorer vaccination response after COVID-19 vaccinations in older persons [30, 31], and Nacash et al. [1] identified age as one of the main predictors of humoral response after SARS-CoV-2 vaccination in ESRD patients. Obesity, on the other hand, was significantly associated with poor HBV non-response, and obesity rates were around 1.5-fold higher in COVID-19 vaccination poor vs. high responders. These findings are in accordance with reports on reduced HBV vaccination responses in obese persons [32]. Also, for SARS-CoV-2 and other pathogens, low vaccination responses have been linked to increased BMI and obesity [33].

While we observe strong concordance of inferior immune responses after HBV and COVID-19 mRNA  vaccination in our study, it is worth considering the separate immunostimulatory pathways HBV and COVID-19 mRNA vaccine employ for induction of humoral immunity. The main adjuvants in the HBV vaccines HbVaxPro and Engerix, are aluminium salt, which stimulate the activation of the NLRP3 inflammasome [34], resulting in the production of IL-1 and eventually local inflammation and recruitment of antigen-presenting cells. The AS04-adjuvant in Fendrix^®^ activates the toll-like receptor (TLR) 4 and induces cytokine production through the NF-κB pathway, which leads to the activation of innate immune cells, but is not a good T cell antigen [20]. In contrast, studies in mice revealed that induction of antibody responses to BNT162b2 is neither dependent on signaling via Toll-like receptors 2, 3, 4, 5 and 7 nor on inflammasome activation [35]. Instead, RNA vaccines induce synthesis of protective virus spike antigen in the target cell itself which, together with a predominantly IL-1β and IL-1 dependent production of a broad spectrum of pro-inflammatory cytokines enhances antigen presentation to T helper cells [21]. Booster vaccination with mRNA vaccines further enhance innate immune response leading to greater frequency of inflammatory monocytes and higher concentrations of plasma IFN gamma [22]. These data are in line with our recent systems biology analysis indicating that the quantity of the adaptive immune response to the BNT162b vaccine are largely determined by the quality of the innate immune response within 24 h after vaccination [36]. In that study we provide evidence that B and T cell responses heavily depend on signals received from the myeloid compartment, specifically on pro-inflammatory cytokines such as IFN. We conclude that dialysis patients have disturbed immune functions affecting pathways targeted by different vaccine platforms. Whether these dysfunction mainly reside in the innate and adaptive immune system remains to be determined.

We were unable to confirm earlier reports on a negative effect of immunosuppressive medication on vaccination responses in chronically ill patients [1, 37] which might be due to our small sample size. Besides this, our study has further limitations. While COVID-19 vaccination response was analyzed prospectively, data on HBV vaccination were extracted retrospectively from patient charts. Given the real-world nature of our data set, there was a significant time period between vaccination against HBV and COVID, and ESRD patients underwent immunization with distinct HBV vaccines. The fact that data were collected retrospectively, may have led to incomplete obtainment of information, and the strategy of excluding analyzing patients with incomplete information about HBV vaccination may have introduced a selection bias. Also, a more comprehensive collection of information on clinical parameters or patient-reported outcomes such as frailty, smoking, alcohol consumption, and other lifestyle factors would have been desirable [15, 32]. Furthermore, clinical history and further information on modifying factors were collected at the timepoint of COVID-19 vaccination and may have changed since the HBV vaccination. However, given the age profiles of our patients, comorbidities such as obesity and diabetes are unlikely to have changed, since trajectories for these diseases usually start earlier in life and are stable throughout adulthood (38).

Despite these limitations, our work delivers important information on vaccination efficacy in the particularly vulnerable population of ESRD hemodialysis patients in a real-life setting. We show that vaccination responses for HBV and COVID-19 display strong overlaps, and that obesity is associated with HBV non-response, while elevated age is linked to reduced SARS-CoV-2 immunity post-vaccination. Health care professionals taking care of ESRD patients should adjust their vaccination strategies when observing an low response after initial vaccinations, e.g., by enhancing post-vaccination antibody surveillance and booster vaccinations in patients that responded poorly to HBV and/or COVID vaccinations. We hope this data helps to improve prevention strategies against communicable diseases in ESRD patients.

## Supplementary Information

Additional file 1.

## Data Availability

The data and biomaterials underlying our analysis may be requested by submitting a formal request to the study board, who will evaluate it. Email requests may be directed to happle.christine@mh-hannover.de.
