# Supplementary material for: Impaired Hepatitis B and COVID-19 vaccination responses show strong concordance in hemodialysis patients with end stage renal disease
Source: Eur J Med Res. 2025 Jan 16;30:34. doi: 10.1186/s40001-025-02274-3 (PMC11736940; doi:10.1186/s40001-025-02274-3)
Supplement: Supplementary file 1 — Additional file 1. [file 40001_2025_2274_MOESM1_ESM.docx]

# Supplementary material

#
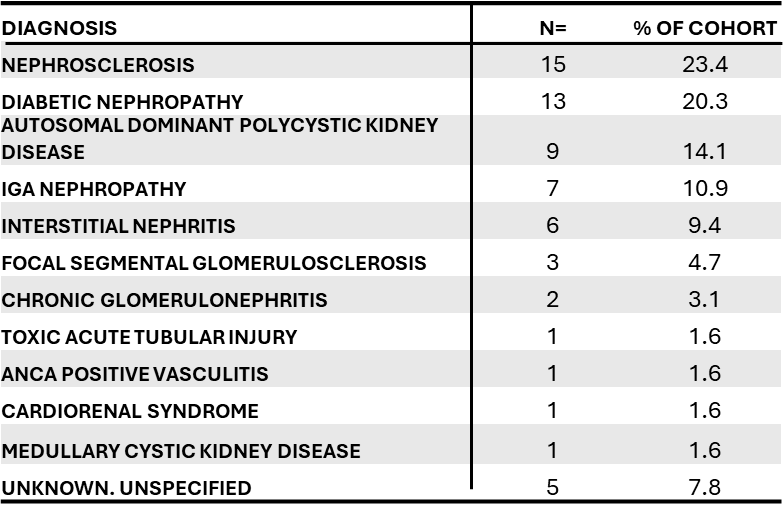


*Suppl. Table 1: Underlying disease frequencies within the cohort of ESRD patients*

*Suppl. Fig. 1: Anti-HBs levels after vaccination with the three vaccine types (graphs display median plus IQR).*

*Suppl. Fig. 2: Correlation of anti-Hbs titres vs. anti-S-IgG within the cohort of ESRD patients.*
